# Essay on Pneumonia

**Published:** 1878-03-20

**Authors:** C. H. Hughes


### ESSAY ON PNEUMONIA.

(Read before the St. Louis Medical Society.)

By Dr. C. H. Hughes.

The discussion of this subject at the two preceding meetings, verifies the statement of a distinguished author that "one of the most discordant topics in the science of medicine seems to be the treatment of pneumonia."

Within a half century the pendulum of professional opinion has swung from the extreme of excessive venesection to that of over-stimulation. The reaction of Laennec and Louis, against indiscriminately breaking the organic structure by withdrawing the material of its reconstruction through the supporting columns of the circulation, was followed in some quarters by the alcoholic insanity of Todd and Bennett. A theoretical delusion based upon the illusory, undemonstrated and undemonstrable view, that because in nearly all cases there exists more or less of oppression and prostration, therefore they are all or nearly all so similarly asthenic in character as to require that kind of stimulation, mainly to be found in alcohol and that sort of nutrition, which exists *par excellenee* in the opinion of some, but in one view, sparingly if at all in uncombined alcohol, that is, in alcohol when not administered in the form of wine, malt liquors, whiskies, brandies, etc.

The crippled and laboring lung and over-acting heart say the alcohol theorists, require to be stimulated. They liken the rapidly beating heart especially to an over-burdened horse, making little progress but very great effort to get up hill, because of the obstructions in his way, which they would remove—the whip and spur, and oats of alcohol.

It seems never to have occurred to these therapeutic logicians, that lightening the load of the jaded animal and conserving his strength, by timely rest and restraint put upon inordinate and futile effort, made at the expense of that latent power upon which the animal's remaining and containing vitality depends, might both save the horse and transport in due time his burden.

Medical theories are often attempts at generalization of morbid states, and therapeutic conclusions drawn from them are frequently misleading in special cases. In fact, for clinical purposes are generally safest.

Theories are the short route flight, which genius makes, rising above rather than overcoming obstacles. The practical physician at the bedside must remove, and not evade, disagreeable morbid obstructions which lie in the way of his patient's recovery.

As a theory the law of *similia* has been tried, and though still in vogue with a large body of theoretical practitioners, as the therapeutic law governing other cases than those wherein the heart is initiated to over-action by morbid excitation, has been found in the light of the general practical observation of the profession, wanting in supporting facts.

In the treatment of pneumonia there are many facts to be considered.

Though closest theorists may ignore them they cannot thus be abolished. It has its variable and varying stages and varieties—age, season, climate, epidemic, endemic influences, and accompanying constitutional cachexias modify its manifestations. Extent of lung tissue involved, the stage at which treatment is begun, as well as the nutrition of the patient preceding, and his surroundings during the attack, so that it cannot be treated as a never varying condition.

The tenants of poverty's abodes, in the back streets and alleys of our crowded cities, who come under treatment at dispensaries and charity hospitals, present not all the same features as the well nourished citizen stricken in the midst of active business, owing to some sudden disturbance in the equilibrium of the circulation, determining a localized inflammation.

Some depression of system from business worry and perhaps deprivation of sleep, often precedes the coming on of these latter cases, but there is seldom mal nutrition and assimilation to any great extent.

Pneumonia with hepatitis, phthisis, typhoid fever, and typhoid states, are not precisely the same as the pneumonitis of traumatism or suppressed perspiration. Yet hospital reports put them forth to sustain a theory, and mass all these dissimilar forms together, as so many illustrative cases, and when the majority recover, as they would under proper hygienic conditions and nursing alone, the advocates of the alcohol plan are exultant in one hospital and those of the expectant are equally elated in another.

One well marked case of double asthenic pneumonia with congestion in every part of both lungs, with a temperature reaching 106°, falling to 100° under ten grain doses of quinine every six hours, with forty or more respirations and a pulse of one hundred and thirty beats per minute, falling under six-drop doses of veratrum viride every four hours, to twenty respirations and a pulse of seventy-five or eighty, is worth more to me than scores of cases recorded without a minute statement of temperature, pulse, respirations, extent of pulmonic surface involved, etc.

I have seen more than one such case, and they are the cases which, without controlling, generally die.

Twenty years ago, before so much attention was paid to temperature in disease as now, and before it was generally conceded that quinine would lower the body heat, I saw such a case conducted to a safe and rapid recovery on veratrum viride alone. The veratrum treatment then made upon me a profoundly favorable impression which has not been effaced by an extensive subsequent experience.

Veratrum viride timely and freely given in the congestive stage, with quinine and Dover's powder, have, in my hands, many times, cut short the progress of pneumonia in its congestive stage. I have tried it in the field, in the hospital and in civil practice.

A large number of cases falling under observation in charity hospitals have generally reached the stage of commencing hepatization in some part of the lung before the patient's admission. I would not expect much from attempts at aborting the disease there.

When hepatization is somewhat advanced or the suppurative stage has been reached, I am willing to compromise with the alcohol advocates on the champagne *pro re rata*.

In the practical management of this disease it is not a never vaiying something which we term pneumonia, that we are to manage, but it is the temperature, the pulse, the respiration, the pain and pulmonic oppression, together with the assault the disease is making on the vital stamina of the patient, that are to be regarded, and these are to be considered according to their varying intensity in all forms and all stages of the disease.

There are some extremely asthenic cases where stimulant quantities of alcohol may do good. There are others where it does little harm, and others which, in spite of the harm it does, recover; and there comes a stage of hepatization and suppuration in many cases when to withhold milk punch or egg-nog would be homicidal. But after all, it is the pulse, the temperature, the respiration, the nutrition, the pangs, the cough, and the nervous shock that are to be controlled and regulated. The remedies which best regulate them are well known, and alcohol, for that stage and kind of pneumonia when we might not trust the disease to nature and nursing, is not the remedy.

The value of cups and poultices for the first stage, and of blisters for advanced second stage, of suitable expectorants and the benefit of a mercurial cathartic in the beginning are conceded. In my army experience, I am confident that I have averted many a pleuro-pneumonia by a brisk cathartic and quinine in full doses during the day and fifteen or twenty grains of Dover's powder at night, and I may say the same of copious hot whisky punch given, when on the march, in lieu of the Dover's powder.

But when a typical sthenic pneumonia is fully established with a pulse above 120, and forty respirations per minute and a temperature of 104° or 106°, who would think of alcohol then as the best remedy?

If such cases recover under alcohol, it must be in spite of it.

I like not the simile of the tired horse, needing only alcoholic whip and spur applied to the excited organism.

The fault is not in the struggling nag—the heart, but in the driver—the excited cardiac and vaso-motor nerve centres responding abnormally to immediate and reflex irritation.

Let us soothe, restrain and retard the animal, rather than goad him to increased exertion beyond his strength.

Gelseminum and digitalis have answered well sometimes in my hands, when the stomach has been irritated.

I could not well dispense with opium, the valerianates, camphor, and the bromides, for their anodyne and hypnotic properties as adjuncts to the sheet anchors, veratrum viride, quinine and nutrients.

I have found use also for Hoffmanns' anodyne, spts. mendereri and nitre, the tinct. of gelseminum acting admirably with the two latter. Belladonna, hyoscyamus, and the fluid extract of wild cherry have a minor place in my therapeutic armamentariam.

I have discarded chloral as an unsafe hypnotic, especially in the second and third stages of pneumonia.—*St. Louis Medical Journal.*

[As a stimulating expectorant and anodyne in these latter stages of pneumonia, we have found the carbonate of amonia combined with morphine, in small doses, oft repeated to answer the purpose.—Ed.]

